# Serum exosomal miR‐378 upregulation is associated with poor prognosis in non–small‐cell lung cancer patients

**DOI:** 10.1002/jcla.23237

**Published:** 2020-02-14

**Authors:** Yan Zhang, Hongjie Xu

**Affiliations:** ^1^ Department of Respiratory Medicine the Fifth People’s Hospital of Wuxi Wuxi City China

**Keywords:** exosomal miR‐378, non–small‐cell lung cancer, prognosis, serum

## Abstract

**Background:**

Deregulated circulating microRNAs (miRNAs) are potential biomarkers for the early detection and prognosis prediction of non–small‐cell lung cancer (NSCLC). The aim of the present study was to investigate the expression pattern of serum exosomal miR‐378 in NSCLC and its correlation with clinical variables.

**Methods:**

Quantitative reverse transcription‐polymerase chain reaction (qRT‐PCR) was performed to detect serum exosomal miR‐378 levels in 103 patients with NSCLC and 60 control subjects.

**Results:**

Our results showed that serum exosomal miR‐378 was significantly overexpressed in NSCLC patients, and serum exosomal miR‐378 upregulation was clearly associated with positive lymph node metastasis and advanced TNM stage. In addition, receiver operating characteristic (ROC) analysis demonstrated that combination of serum exosomal miR‐378 expression and carcinoembryonic antigen (CEA) had a high discriminating power to differentiate NSCLC subjects from controls. Moreover, serum exosomal miR‐378 levels in 73 NSCLC cases were significantly decreased after radiotherapy and could be used as an indicator of radiotherapeutic response in NSCLC. Furthermore, survival analyses revealed that patients with higher serum exosomal miR‐378 expression had poor overall survival. Multivariate analysis showed that serum exosomal miR‐378 expression was independently associated with overall survival.

**Conclusions:**

Collectively, serum exosomal miR‐378 has strong potential as a promising non‐invasive biomarker for screening and monitoring NSCLC.

## 1 INTRODUCTION

Lung cancer is the most frequent leading cause of cancer‐related death all around the world, and about 60 000 patients with lung cancer die every year in China. The majority of lung cancer (approximately 85%) are diagnosed as non–small‐cell lung cancer (NSCLC), and tumor metastasis is found in most NSCLC cases at diagnosis.^1^, ^2^ Although great advancements in the therapies such as radiation and chemotherapy have been made over the past decades, the 5‐year survival rate of this malignancy remains unfavorable. Early diagnosis and accurate prognosis prediction can reduce the mortality of this disease and improve patients’ survival time.^3^, ^4^ Therefore, it is of importance to identify novel and reliable biomarkers for early detection and prognosis prediction of NSCLC.

Exosomes are small, cell‐secreted vesicles with a diameter about 30‐100 nm which carry proteins, RNAs, miRNAs, or lipids. Exosomes are widespread in the blood, serum, urine, and other bodily fluids.^5^, ^6^ MicroRNAs (miRNAs) are mall non‐coding RNAs which post‐transcriptionally regulate protein expression by binding to the 3′‐UTR region of mRNAs. MiRNAs can function as tumor suppressors or oncogenes by regulating the expression of cancer‐related gene and are associated with the tumorigenesis and development of many cancers.^7^, ^8^ Previous studies have showed that most of serum miRNAs are enriched in exosomes and stably detected.^9^ Thus, serum exosomal miRNAs can be used as potential biomarkers for the diagnosis and prognosis of many cancer types, including NSCLC.

MiR‐378 is located on chromosome 5q32, and its role in progression of NSCLC has been explored by some previous studies. Ji and colleagues showed that miR‐378 expression was markedly higher in lung cancer tissues than in non‐cancerous tissues, and miR‐378 underexpression inhibited cancer cell proliferation and induced cell apoptosis by targeting FOXG1, RBX1, and CRKL.^10^, ^11^ Similarly, Skrzypek et al reported enforced miR‐378 expression or HMOX1 downregulation significantly promoted cancer cell proliferation and migration in vitro and in vivo*,* and decreased miR‐378 expression exerted opposite effects.^12^ However, the clinical significance of serum exosomal miR‐378 as a biomarker in patients with NSCLC remains poorly known. In this study, we detected the serum exosomal miR‐378 expression levels in 103 NSCLC patients and assessed its potential value for NSCLC diagnosis and prognosis.

## 2 MATERIALS AND METHODS

### 2.1 Patients and serum sample preparation

A total of 103 patients diagnosed with NSCLC, 32 patients with non‐malignant respiratory diseases (NMRD), and 60 healthy volunteers were recruited in our study. The NSCLC patients receiving any radiotherapy or chemotherapy prior to blood sample collection were excluded. The group comprised 62 men and 41 women, with a median age of 57.4 years (range 26.5–69.3). The tumor‐node‐metastasis (TNM) classification for staging of NSCLC was in accordance with the 8th edition of TNM staging system. Patient details, including sex, age, histology, tumor size, differentiation, lymph node metastasis, and TNM, were retrospectively collected (Table 1). For the cases with NMRD, thirteen patients were diagnosed with bronchiectasis and the remaining 19 patients were diagnosed with chronic obstructive pulmonary disease. The study was approved by the Ethics Committee of the Fifth People's Hospital of Wuxi, and written informed consent was collected from each participant before sample collection.

**Table 1:** Clinicopathological correlation of serum exosomal miR‐378 expression in NSCLC patients

Characteristics	Patients (N = 103)	Serum exosomal miR‐378		*P*
		Low	High	
Sex				
Man	62	25	37	.184
Woman	41	22	19	
Age				
<60	47	21	26	.859
≥60	56	26	30	
Histology				
Adenocarcinoma	53	23	30	.473
Squamous cell carcinoma	46	21	25	
Others	4	3	1	
Tumor size				
<5 cm	74	36	38	.326
≥5 cm	29	11	18	
Differentiation				
Well	11	8	3	.131
Moderate	53	24	29	
Poor	39	15	24	
Lymph node metastasis				
Negative	68	37	31	.013
Positive	35	10	25	
Tumor‐node‐metastasis				
I‐II	63	36	27	.003
III‐IV	40	11	29	

Up to 5 mL blood samples were withdrawn from all the participants and stored in tubes containing ethylenediaminetetraacetic acid (EDTA). Moreover, paired serum samples from 73 NSCLC patients treated with radiotherapy were collected six weeks later. The samples were separated by centrifugation at 1600 *g* for 10 minutes. Subsequently, the supernatant sera were divided into small aliquots and stored at −80°C.

### 2.2 Exosome isolation

The exosomes were isolated from the serum samples using ExoQuick™ Kit (System Biosciences, Palo Alto) according to the manufacturer's instructions. Briefly, the serum samples were centrifuged at 12 000 *g* for 5 minutes. Then, the supernatant was mixed with ExoQuick™ solution. Follow by incubation at 4°C for 30 minutes, the mixture was centrifuged at 2000 *g* for 30 minutes, and the exosome was resuspended in nuclease‐free water. The exosomes were stored at −80°C until further use.

### 2.3 Extraction of RNA and quantitative reverse transcription‐polymerase chain reaction (qRT‐PCR)

The total RNA was isolated from serum exosomes using the Qiagen miRNeasy Kit (Qiagen). The quantity and purity of the isolated RNA were evaluated using a NanoDrop ND‐1000 Spectrophotometer (NanoDrop). The cDNA was reverse‐transcribed using the TaqMan™ MicroRNA Reverse Transcription Kit (Thermo Fisher Scientific, Inc). qRT‐PCR was carried out using an 7900HT Fast Real‐Time PCR System (Applied Biosystems) according to the manufacturer's instructions. During RNA isolation, 25 fmol of synthetic cel‐miR‐39 was added as a spike‐in control miRNA into each sample. The serum exosomal miR‐378 expression level was evaluated using the 2^−ΔΔCt^ method, and each experiment was performed in triplicate. The expression levels of serum exosomal miR‐378 were compared between patients with NSCLC and healthy individuals (2^−(Ct[miR‐378] −Ct[cel‐miR‐39]) NSCLC−(Ct[miR‐378]−Ct[cel‐miR‐39]) CTRL^), patients with NSCLC and patients with NMRD(2^−(Ct[miR‐378]−Ct[cel‐miR‐39]) NSCLC−(Ct[miR‐378]−Ct[cel‐miR‐39]) NMRD^), patients with NMRD, and healthy individuals (2^−(Ct[miR‐378]−Ct[cel‐miR‐39]) NMRD−(Ct[miR‐378]−Ct[cel‐miR‐39]) CTRL^).

### 2.4 Statistical analysis

All statistical analyses were performed by GraphPad Prism (GraphPad Software) and MedCalc 16.7 (MedCalc). The Kruskal‐Wallis test was conducted to compare the serum exosomal miR‐378 levels among three compared groups. Differences between serum exosomal miR‐378 expression and clinicopathological parameters were analyzed using the chi‐square test. Receiver operating characteristic (ROC) curves were applied to assess the diagnostic values of serum exosomal miR‐378 and CEA, and the value of area under the curve (AUC) was calculated. Overall survival (OS) was calculated from the date of NSCLC diagnosis to the date of death or last follow‐up. Survival analysis was performed using the Kaplan‐Meier method plus log‐rank test. Multivariate analyses of OS were performed using a Cox proportional hazards model. *P *value of <.05 was considered as statistically significant.

## 3 RESULTS

### 3.1 Upregulation of serum exosomal miR‐378 in NSCLC samples

**Figure 1:**
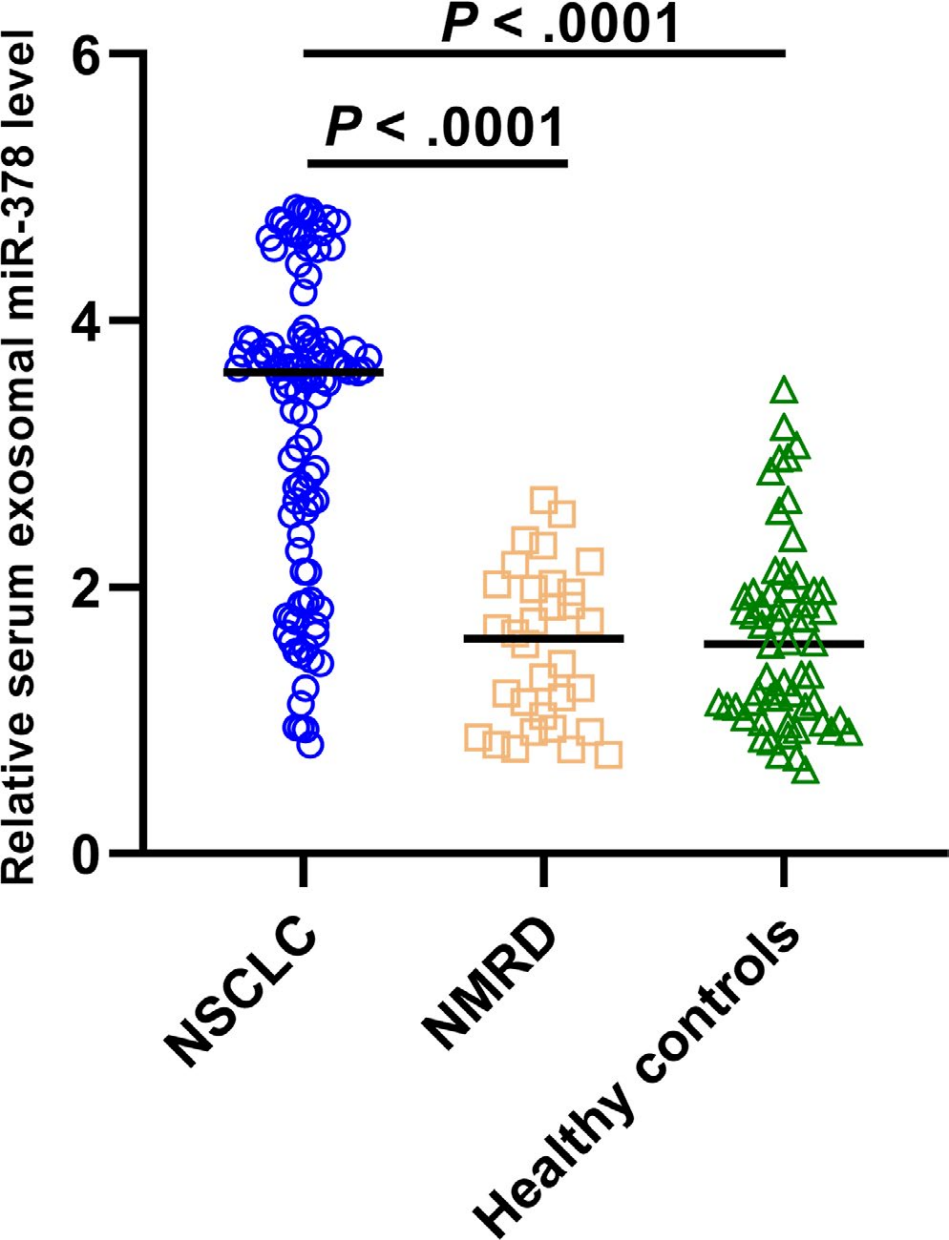
Serum exosomal miR‐378 levels were higher in NSCLC patients than in NMRD patients and healthy controls

Real‐time PCR was performed to measure the expression levels of serum exosomal miR‐378 in different groups. As shown in Figure 1, serum exosomal miR‐378 levels were significantly higher in NSCLC subjects compared to the patients with NMRD (*P* < .0001) and the healthy controls (*P* < .0001). No significant difference was found for the serum exosomal miR‐378 level between healthy individuals and patients with NMRD.

### 3.2 Relationship between serum exosomal miR‐378 level and clinical features of NSCLC

Then, the association between serum exosomal miR‐378 expression level and the clinical features of NSCLC was explored. All patients were divided into high and low expression groups based on the median expression levels of serum exosomal miR‐378. According to the criterion, 56 cases and 47 cases were classified as high and low expression groups, respectively. As illustrated in Table 1, increased serum exosomal miR‐378 expression occurred more frequently in tumors with positive lymph node metastasis (*P* = .013) and advanced TNM stage (*P* = .003), indicating that miR‐378 might involve in the progression of NSCLC. However, there was no significant association of serum exosomal miR‐378 expression with patients' sex (*P * = .184), age (*P * = .859), histology (*P * = .473), tumor size (*P * = .326), or differentiation (*P * = .131).

### 3.3 Diagnostic implication of serum exosomal miR‐378 and CEA

**Figure 2:**
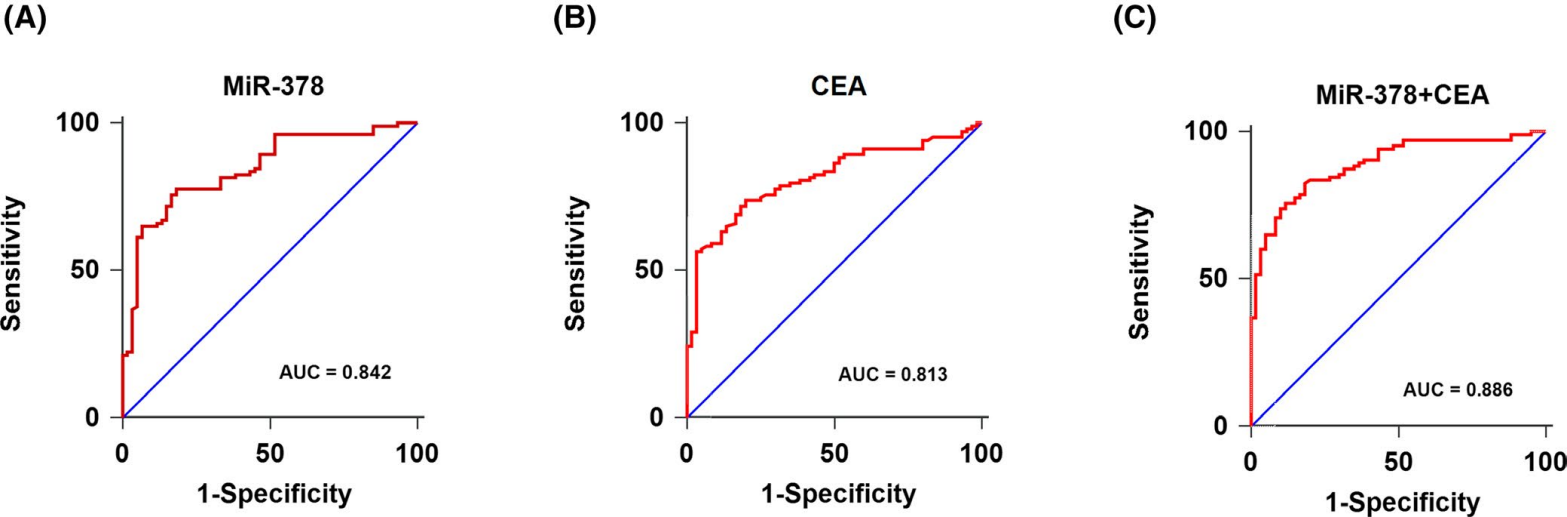
A, ROC analysis for distinguishing NSCLC cases from controls using serum exosomal miR‐378. B, ROC analysis for distinguishing NSCLC cases from controls using CEA. C, ROC analysis for distinguishing NSCLC cases from controls using combination of serum exosomal miR‐378 and CEA

Receiver operating characteristic (ROC) curves were constructed to assess the diagnostic accuracy of serum exosomal miR‐378 and carcinoembryonic antigen (CEA), which was widely used as a serum tumor marker. ROC analysis showed that serum exosomal miR‐378 and CEA were both reliable biomarkers for differentiating NSCLC from normal controls with AUC of 0.842 (Figure 2A, specificity: 81.5%, sensitivity: 77.7%) and 0.813 (Figure 2B, specificity: 80.0%, sensitivity: 73.8%), respectively. Moreover, the combination of serum exosomal miR‐378 and CEA yielded the highest AUC value of 0.886, with 84.7% specificity and 82.5% sensitivity (Figure 2C). The data showed that combination of these two markers was more specific and sensitive in classifying NSCLC from healthy control samples than single marker alone.

### 3.4 Dynamics of serum exosomal miR‐378 level as a predictor of radiotherapeutic response in NSCLC

**Figure 3:**
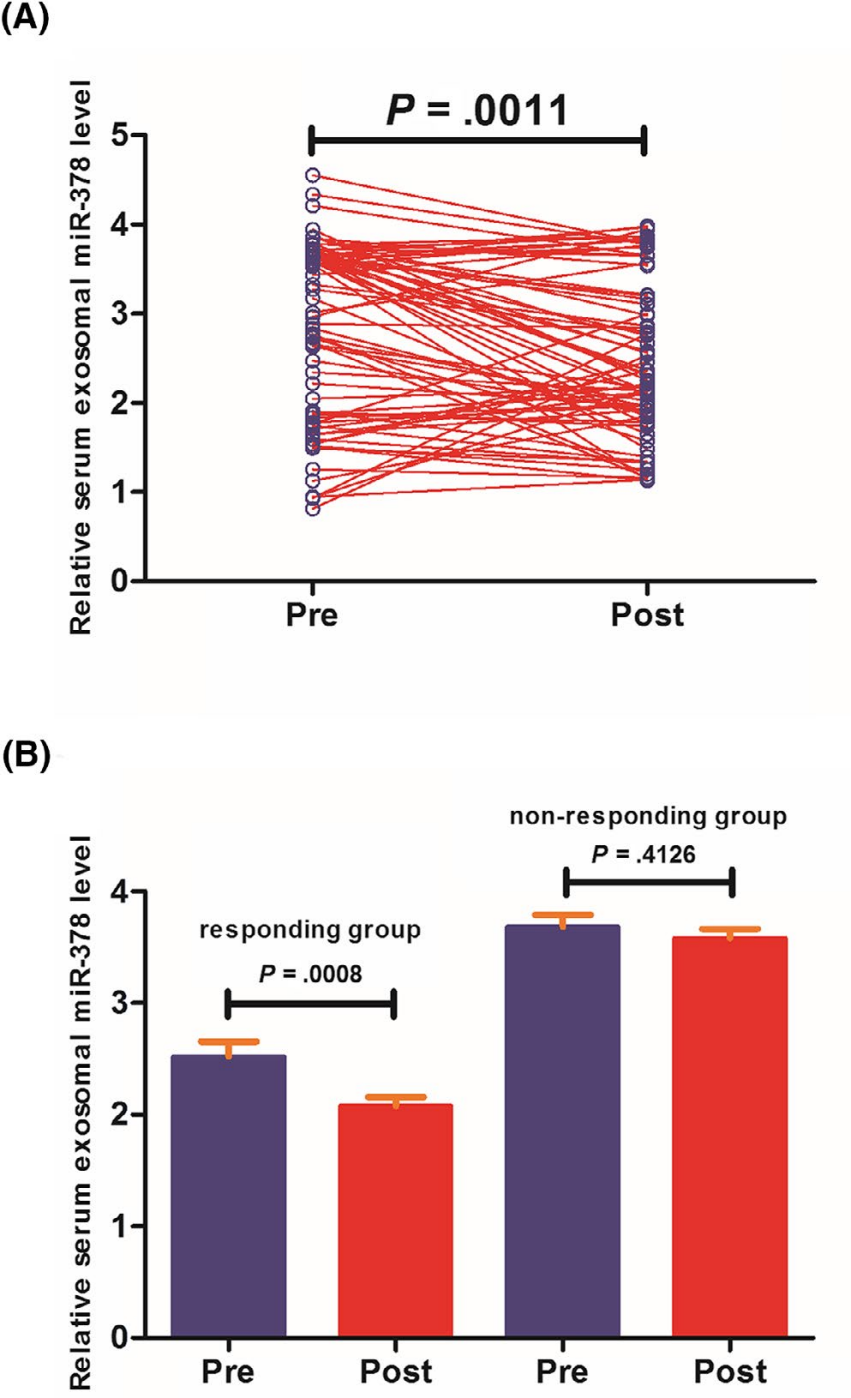
A, Serum exosomal miR‐378 levels in post‐treatment samples were significantly lower than those in paired pre‐treatment samples. B, Serum exosomal miR‐378 levels were significantly downregulated in responding and non‐responding groups after radiotherapy

To assess whether radiotherapy affected serum exosomal miR‐378 expression, the pre‐ and post‐radiotherapy serum exosomal miR‐378 levels were compared in 73 patients who received radiotherapy. We found that the expression levels of serum exosomal miR‐378 were greatly reduced in post‐radiotherapy samples compared to those in matched pre‐radiotherapy samples (*P* = .0011; Figure 3A). According to the radiotherapeutic response, 55 patients were classified into responding group (complete remission or partial response), and 18 patients were classified into non‐responding group (stable disease or progressive response). A comparison of serum exosomal miR‐378 levels before and after radiotherapy showed that serum exosomal miR‐378 levels were remarkably decreased in responding group (*P* = .0008; Figure 3A). However, no significant difference was found in non‐responding group (*P* = .4126; Figure 3B).

### 3.5 Relationship between serum exosomal miR‐378 level and NSCLC prognosis

**Figure 4:**
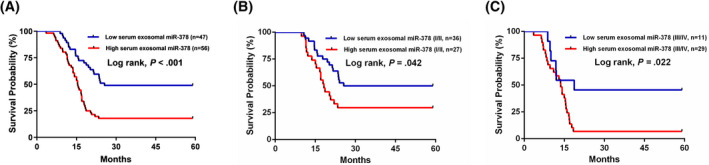
A, Survival curve of OS rates in all NSCLC patients on the basis of high or low serum exosomal miR‐378 levels. B, Survival curve of OS rates in I/II stage NSCLC patients on the basis of high or low serum exosomal miR‐378 levels. C, Survival curve of OS rates in III/IV stage NSCLC patients on the basis of high or low serum exosomal miR‐378 levels

Follow‐up data were available for all NSCLC subjects, and Kaplan‐Meier survival analysis with log‐rank test was used to explore the associations between serum exosomal miR‐378 expression levels and OS. Patients in the high serum exosomal miR‐378 expression group had significantly worse outcomes compared with patients in the low serum exosomal miR‐378 expression group (*P* < .001; Figure 4A). For TNM stage I/II or III/IV patients, patients in high serum exosomal miR‐378 expression group had shorter OS than those in low serum exosomal miR‐378 expression group (*P* = .042; Figure 4B and *P* = .022; Figure 4C).

In a multivariate analysis, increased serum exosomal miR‐378 expression retained prognostic significance for OS (RR = 3.95, 95% CI = 1.71‐6.26, *P* = .024). In addition, positive lymph node metastasis (RR = 4.14, 95% CI = 1.80‐6.62, *P* = .017) and advanced TNM (RR 4.38, 95% CI 1.93‐6.98, *P* = .009) were also independent prognostic indicators for OS (Table 2).

**Table 2:** Multivariate analysis of prognostic factors in NSCLC patients

Features	Risk ratio	95% CI	*P*‐value
Sex	1.35	0.85‐1.97	.163
Age	1.24	0.72‐1.83	.148
Histology	2.56	1.24‐4.02	.108
Tumor size	2.87	1.36‐4.48	.086
Differentiation	3.16	1.55‐4.93	.071
Lymph node metastasis	4.14	1.80‐6.62	.017
Tumor‐node‐metastasis	4.38	1.93‐6.98	.009
Serum exosomal miR‐378	3.95	1.71‐6.26	.024

## 4 DISCUSSION

Non–small‐cell lung cancer is still one of the leading causes for cancer‐related death in China. In this study, we found serum exosomal miR‐378 levels were significantly higher in NSCLC cases than in the patients with NMRD as well as in healthy controls. In addition, elevated serum exosomal miR‐378 levels were strongly associated with unfavorable clinical parameters. Moreover, serum exosomal miR‐378 expression showed good performance to differentiate NSCLC patients from healthy controls. The combination of serum exosomal miR‐378 and CEA improved the specificity and sensitivity for detection of NSCLC, and represented a promising screening test for NSCLC diagnosis. Furthermore, serum exosomal miR‐378 levels were significantly downregulated after radiotherapy and could be used to monitor radiotherapeutic responses in NSCLC patients. Kaplan‐Meier analysis demonstrated patients in high serum exosomal miR‐378 expression group had shorter OS in comparison with those in low serum exosomal miR‐378 expression group. Finally, high serum exosomal miR‐378 expression was confirmed to be an independent poor prognostic marker for NSCLC. These results suggested the serum exosomal miR‐378 might serve as a promising biomarker for the early diagnosis and prognostic prediction of NSCLC.

Previous studies have demonstrated that cancer cells produce and secrete many more exosomes than normal proliferating cells.^13^, ^14^ Therefore, most circulating exosomes in the serum samples of NSCLC patients might be originated from the tumor cells. It is possible that the lung cancer cells derived exosomes (from NSCLC patients) have significantly more miR‐378 than normal cells derived exosomes (from healthy individuals or patients with NMRD), resulting in the upregulation of serum exosomal miR‐378 in patients with NSCLC compared with healthy individuals or patients with NMRD. However, in addition to lung cancer cells, exosomes can be released by most cells of the tumor microenvironment such as cancer associated fibroblasts and immune cells. Exosomal miR‐378 secreted by cancer associated fibroblasts and/or immune cells might be also markedly higher than those released by normal cells, contributing to dramatically increased level of serum exosomal miR‐378 in NSCLC patients. To elucidate whether the miR‐378 is higher in lung cancer cell‐derived exosome, the tumor‐specific exosomes need to be isolated and purified from the serum samples of NSCLC patients. Then, the level of miR‐378 in circulating tumor cell‐ and normal cell‐derived exosomes should be compared.

Likewise, the dysregulation of miR‐378 was reported to have an oncogenic role in various cancer types. In cervical cancer (CC), miR‐378 was overexpressed in CC tissues and cell lines, and in vitro and in vivo evidence showed that miR‐378 upregulation significantly promoted cell growth, migration, invasion, and restrained apoptosis. Both ST7L and ATG12 were its downstream target genes.^15^, ^16^ Zhou et al found that miR‐378 expression was greatly increased in cholangiocarcinoma. MiR‐378 upregulation enhanced the carcinogenesis and was associated with poor prognosis.^17^ In osteosarcoma, upregulation of miR‐378 occurred more frequently in both cancerous tissues and cell lines. Ectopic expression of miR‐378 greatly increased cell proliferation by inversely regulating KLF9.^18^ In melanoma, Sun et al found that miR‐378 expression was significantly upregulated in cancerous tissues, miR‐378 overexpression markedly stimulated oncogenic behaviors of cancer cells both in vitro and in vivo through regulating FOXN3.^19^ Ma et al reported that miR‐378 upregulation significantly promoted the proliferation, migration, and invasion capacity of liver cancer cells by downregulating Fus expression.^20^

Interestingly, accumulating evidence has demonstrated that miR‐378 also played a tumor‐suppressive role in many cancer types. For instance, miR‐378 was downregulated in CRC cancer tissues and cell lines, and miR‐378 upregulation was negatively correlated with aggressive clinical variables. Overexpression of miR‐378 suppressed the malignant behaviors of cancer cells in vitro by targeting CDC40 and vimentin.^21^, ^22^ Similarly, Zeng et al^23^ showed that miR‐378 expression was frequently downregulated both in colon cancer tissues and in cell lines. Enforced expression of miR‐378 not only restrained cancer cell proliferation, but also suppressed carcinogenesis by directly targeting SDAD1. In prostate cancer, decreased miR‐378 expression was observed in cancer tissues and predicted poor prognosis. Ectopic expression of miR‐378 significantly attenuated cancer cell migration, invasion, and stimulated cell apoptosis.^24^, ^25^ In addition, Fei and colleagues showed enforced miR‐378 expression remarkably repressed gastric cancer cell proliferation, cycle progression, migration, and invasion by negatively regulating MAPK1^26^. Moreover, Li et al showed miR‐378 expression was dramatically decreased in glioma tissues compared to adjacent normal tissues, and low miR‐378 expression was associated with shorter overall survival.^27^ These data suggested that miR‐378 might act as either an oncomiR or tumor suppressor gene in cancer. The biological functions of miRNAs might largely depend on its downstream targets. Therefore, it is reasonable to observe the phenomenon that miR‐378 plays a contradictory role in different types of cancers.

In summary, we have revealed an increase in serum exosomal miR‐378 levels in NSCLC patients. The combination of serum exosomal miR‐378 and CEA represented a very promising screening test for NSCLC diagnosis, and higher serum exosomal miR‐378 levels were associated with unfavorable prognosis of NSCLC. Collectively, serum exosomal miR‐378 might be a reliable biomarker for the diagnosis and prognosis of NSCLC.

## Data Availability

The data that support the findings of this study are available on request from the corresponding author.
